# Effect of preharvest conditions on cut-flower quality

**DOI:** 10.3389/fpls.2023.1281456

**Published:** 2023-11-10

**Authors:** Julian C. Verdonk, Wim van Ieperen, Dália R. A. Carvalho, Geert van Geest, Rob E. Schouten

**Affiliations:** ^1^Department of Horticulture and Product Physiology, Wageningen University and Research, Wageningen, Netherlands; ^2^Rijk Zwaan Breeding B.V., DeLier, Netherlands; ^3^Interfaculty Bioinformatics, Institut für Biologie, Fakultät für Naturwissenschaften und Naturwissenschaften, Universität Bern, Bern, Switzerland; ^4^Wageningen Food & Biobased Research, Wageningen University and Research, Wageningen, Netherlands

**Keywords:** vase life, ornamental crops, pathogens, water balance, stomata, xylem architecture, carbohydrate starvation

## Abstract

The cut flower industry has a global reach as flowers are often produced in countries around the equator and transported by plane or ship (reefer) mostly to the global north. Vase-life issues are often regarded as linked to only postharvest conditions while cultivation factors are just as important. Here, we review the main causes for quality reduction in cut flowers with the emphasis on the importance of preharvest conditions. Cut flower quality is characterised by a wide range of features, such as flower number, size, shape, colour (patterns), fragrance, uniformity of blooming, leaf and stem colour, plant shape and developmental stage, and absence of pests and diseases. Postharvest performance involves improving and preserving most of these characteristics for as long as possible. The main causes for cut flower quality loss are reduced water balance or carbohydrate availability, senescence and pest and diseases. Although there is a clear role for genotype, cultivation conditions are just as important to improve vase life. The role of growth conditions has been shown to be essential; irrigation, air humidity, and light quantity and quality can be used to increase quality. For example, xylem architecture is affected by the irrigation scheme, and the relative humidity in the greenhouse affects stomatal function. Both features determine the water balance of the flowering stem. Light quality and period drives photosynthesis, which is directly responsible for accumulation of carbohydrates. The carbohydrate status is important for respiration, and many senescence related processes. High carbohydrates can lead to sugar loss into the vase water, leading to bacterial growth and potential xylem blockage. Finally, inferior hygiene during cultivation and temperature and humidity control during postharvest can lead to pathogen contamination. At the end of the review, we will discuss the future outlook focussing on new phenotyping tools necessary to quantify the complex interactions between cultivation factors and postharvest performance of cut flowers.

## Introduction

Vase life of cut flowers is the time flowers have a good appearance. Vase life depends on genotype, preharvest and postharvest conditions. End of vase life can be determined by discolouration of flowers, yellowing of leaves, wilting of leaves or flowers, bending of stems, abscission of flower-(petals) or leaves, presence of pathogens, turbid vase water, inflorescence blackening, drooping of the flower head, and often a combination of these processes (van Meeteren, 1992; van Doorn and Cruz, 2000). Although breeding has led to a spectacular variety in phenotypes, improving postharvest keeping quality is still a challenge. This is because harvesting leads to dramatic changes in environment; especially when plant products such as cut flowers, are transported and/or stored. Temperature, light, CO_2_ level, air velocity and humidity change considerably, influencing photosynthesis, respiration, and transpiration.

The carbohydrate level of cut-flowers can be strongly influenced by cultivar, growth practices and postharvest treatments (Zieslin et al., 1975; Rajapakse and Kelly, 1995; Eason et al., 1997; Han, 2003; Kazuo et al., 2005; van Geest et al., 2017). A negative carbohydrate balance may lead to carbohydrate starvation, which may lead to induction of senescence. Hormone levels have a striking effect on flower longevity and senescence; important hormones such as ethylene, gibberellins, auxins, and abscisic acid have been implicated in flower longevity and keeping quality (Reid and Jiang, 2012). For example, ethylene can lead to flower senescence and leaf abscission, in climacteric species such as carnations and rose. Harvest is the onset of potential water loss and a reduction of water content in cut flowers (Halevy and Mayak, 1979; van Doorn, 1996). This occurs through continued transpiration through stomata: openings between two guard cells that control gas exchange between inner and outer environment of the plant. To prevent water loss, stomata have to close, triggered by environmental conditions (Fanourakis et al., 2013b). Pathogen pressure can shorten vase-life greatly, by negatively affecting the water balance, e.g., by bacteria in vase water (van Doorn, 1996). In addition, various phytopathogenic fungi, amongst others, *Botrytis cinerea* is an ever-present disaster risk that creates leaf spots, blight in leaves, stems, and flowers, sepal yellowing, and peduncle bending (Bika et al., 2021).

This mini review aims to provide an overview of the effects of cultivation factors on the (postharvest) quality of cut flowers. Better understanding of these effects will be useful to grow higher quality cut flowers and speed up the selection of new genotypes with improved quality characteristics. The aim of this mini review is not to describe cut flower quality issues in detail as many other reviews have described these in great detail and are referenced in the text where relevant.

## Water balance

In cut flowers, the negative effects of water loss through transpiration are large when water uptake is limited. When a flower is cut, the water content of the flowering stem is at risk: water uptake through xylem is obstructed whereas evaporation through stomata may continue (Fanourakis et al., 2013b; Schouten et al., 2018a). Water content is an important factor for vase life of cut flowers. The water content is commonly measured by following the fresh weight over time, as well as the water uptake and transpiration of water (van Meeteren et al., 1999). Although end of vase life is not only dependent on water content, end of vase life is often correlated to the point where fresh weight drops below a certain threshold of the initial fresh weight (van Meeteren, 1992; van Meeteren et al., 1999). Genotype and environment during preharvest and postharvest are both important. The effect of preharvest factors such as harvest date, and cultivation conditions determine water balance between uptake and transpiration (Halevy and Mayak, 1979; Torre and Fjeld, 2001; van Meeteren et al., 2005).

Water transport occurs through the xylem: a water transport network of long, dead, tracheary cells that facilitates mostly transpiration but also is important maintaining the water content in the stem. It consists of vessels and tracheid, both interconnected through perforations in their common walls. The interconnection is achieved through pit pores; thinner portions of the cell wall that allow fluids movement. The rate of water transport, the hydraulic conductance (K_h_), is proportional to the radius of the vessel (Zimmermann, 1983; van Doorn, 1996; van Doorn, 2012). Because water transport is facilitated by the xylem, blockage and resistance to water flow, can lead to a negative water balance and subsequent wilting (Aarts, 1957; Halevy and Mayak, 1979; Halevy and Mayak, 1981). Blockage occurs through microbial growth in the vase water, the occurrence of air emboli, or a wound response.

Microbial growth in the vase water causes xylem blockage limiting water uptake leading to premature wilting: addition of bacteria into vase water shortened vase life (van Doorn, 1996; Schouten et al., 2018b). Vase water bacteria likely originate from the hairy surface of the stem, and take advantage of carbohydrates that leak from wounded surfaces induced by harvesting and postharvest removal of leaves and thorns (Woltering, 1987; Put, 1990). Xylem architecture determines bacterial colonization as bacteria cannot pass the pit pores. Therefore, vessel length is a limiting factor. In rose, bacteria did not travel further than 50 mm from the cut end of the stem (Robinson et al., 2007).

Air emboli are the result of air entering the xylem at the cut end (van Doorn, 1990; van Meeteren, 1992) or higher up in the xylem caused by cavitation which results from strong negative xylem pressure due to strong transpiration. During harvest, the xylem vessels are cut from the root, exposing them to the air, which is sucked into the vessels due to the negative pressure in the xylem. This causes complete embolization. Air does not pass to adjacent non-cut xylem vessels because air cannot pass the pit membranes. The air is trapped between the entering water column and the pit membranes and high surface tension prevent water transport (Zimmermann, 1983; Nijsse et al., 2000; Nijsse et al., 2001; van Ieperen et al., 2001). Large diameter xylem vessels are more susceptible to air emboli than narrow ones. Smaller, less developed vessels are less susceptible to air embolism because they have smaller pit pores (Sperry and Tyree, 1988; Hargrave et al., 1994; van Ieperen et al., 2002). Cutting of the stem under water is the best way to prevent air emboli. Cold water is also advised, because it reduces bacterial growth, and air has a higher solubility in cold water (van Meeteren, 1992). Reducing the surface tension by addition of surfactant can also reduce air emboli (van Doorn, 2012; Schouten et al., 2018a).

Mechanical damage of the tissue can induce a wound response: the deposition of materials such as gums and mucilage in the lumen of xylem vessels, and formation of tyloses on the cut stem surface (van Doorn, 1996). These water-uptake restricting depositions are considered a physiological (oxidative) wound response to cutting, depending both on peroxidase and catechol oxidase activity. Inhibitors of oxidative enzymes can prevent these responses (van Doorn and Cruz, 2000; Çelikel et al., 2011).

Removal of 2.5 to 5 cm of the stem improves water balance, removing air emboli, bacterial colonization, and wound induced depositions. However, there is hesitance to remove too much because stem length determines value (Moody et al., 2014). Also, further wounding of the tissue could lead to the additional biosynthesis of blocking depositions. Commercial vase solutions contain carbohydrates to prevent early senescence, and in addition, compounds that prevent bacterial growth, air emboli and wound response (Vaslier and van Doorn, 2003).

## Role of cultivation on xylem architecture

For most cut flower quality related factors, the xylem architecture is important. Although wide vessels are responsible for most of the water transport, the presence of narrow shorter vessels is needed to overcome air emboli (Zimmermann, 1983; van Doorn, 1996). In grape, it was shown that bacterial growth is restricted in cultivars with more narrow xylem vessels (Chatelet et al., 2011). Nevertheless, dedicated experiments linking growth conditions with bacterial xylem blockage have not been carried out, perhaps due to the complexity of the interacting factors that not only affect the risk of cavitation but also affect the type of bacteria growing on stems (Carlson et al., 2015). Experiments with varying light intensity, and cultivation temperatures led to only minor changes in the ability to restore K_h_ after induction of air emboli. This was observed when conditions were rather extreme (van Meeteren et al., 2005). However, the water content of the root substrate was shown to have a significant effect on restoration of induced air emboli in chrysanthemum flowering stems. Without air emboli, stems grown at perlite with a 70% water content had a better K_h_ that those grown at 20% (van Meeteren et al., 2005). In grape and zinnia, the effect of reduced irrigation and water stress during growth was also shown to lead to reduced xylem diameter (Lovisolo and Schubert, 1998; Twumasi et al., 2005). This might be employed to optimize water transport during postharvest. However, effect of growth conditions on xylem architecture is anecdotal and limited to few species. The limiting factor is quick and non-destructive quantification of the xylem architecture. Recent work has demonstrated the visualization of xylem architecture with micro-CT images and ultrasound (Schneider et al., 2021; Wason et al., 2021; Dutta et al., 2022). Also helpful is that the xylem architecture can be simulated using water flow equations (Nijsse et al., 2001; van Ieperen et al., 2002; Couvreur et al., 2021). These new non-destructive phenotyping methods can be used to study plants grown under highly controlled growing conditions, allowing the study of the effects of cultivation on xylem architecture (Rosenqvist et al., 2019; van Delden et al., 2021).

## Stomata

A stoma consists of two guard cells that control opening and closing of the stomatal pore by swelling and shrinking, respectively (Schroeder et al., 2001a). Stomata form the connection between internal leaf space and aerial environment around the leaf controlling CO_2_ entry for photosynthesis and outflow of transpired water (Tallman, 2004). Stomatal guard cells are regulated by signals as diverse as light (spectrum, intensity, and photoperiod), relative air humidity (RH), temperature, air velocity, nutrition, leaf water status, and carbon dioxide concentration (Tallman, 2004; Reynolds-Henne et al., 2010; Feller and Vaseva, 2014). In addition, stomatal functioning is also regulated through abscisic acid (ABA) and nitric oxide (NO), and mediated by gene expression and protein activity (Assmann, 1993; Schroeder et al., 2001a; Schroeder et al., 2001b; Cutler et al., 2010; Kline et al., 2010; Assmann and Jegla, 2016; van Meeteren et al., 2020). Recent work in arabidopsis suggests an involvement of ethylene in stomata response, by tuning/accelerating stomatal conductance responses to CO2 and ABA (Azoulay-Shemer et al., 2023).

To reduce energy use in greenhouses, growers are stimulated to grow plants at high RH (>85% RH) (de Gelder and Dieleman, 2012; de Gelder et al., 2012; Marcelis et al., 2014). Growing at high RH often leads to malfunctioning stomata, meaning that they do not respond well to closing stimuli such as light-dark transition, ABA, or desiccation (Torre and Fjeld, 2001; Rezaei Nejad and van Meeteren, 2006; Fanourakis et al., 2011; Arve et al., 2013; Fanourakis et al., 2013a; Aliniaeifard et al., 2014). The consequence of this stomatal malfunctioning is a high transpiration rate after harvest leading to early onset of wilting (Lange et al., 1971; Fanourakis et al., 2012; Arve et al., 2013). A significant shortening (9-80%) of vase life in roses, bouvardia, and chrysanthemum was observed (Torre and Fjeld, 2001; Fanourakis et al., 2013a; Aliniaeifard and van Meeteren, 2016; Fanourakis et al., 2016; van Meeteren and Aliniaeifard, 2016; Aliniaeifard and Van Meeteren, 2018; Schouten et al., 2018a). Interestingly, depending on the species, exposure time to high RH leading to stomatal malfunctioning can vary from less than one day to more than four days (Aliniaeifard and van Meeteren, 2013). There is genotypic variation in stomatal malfunctioning, at least in arabidopsis and rose (Aliniaeifard and van Meeteren, 2014; Carvalho et al., 2016). In arabidopsis, there is a variation in sensitivity to growth at high RH; and in rose, a segregating population was phenotyped for stomatal responsiveness to which led to the identification of genomic regions (Carvalho et al., 2015). This suggests that breeding can be used to reduce the effects of stomatal malfunction.

Stomata respond strongly to light. Continuous light can worsen the negative effects of high RH in stomatal functioning in rose (Mortensen and Gislerød, 1999). High light can alleviate the stomata function, cultivation at PPFD of >200 μmol·m^-2^·s^-1^ led to more responsive stomata and decreased cuticular permeability (Fanourakis et al., 2019). It was shown that a dark period is important to develop functional stomata (Arve et al., 2013). Also, in well-watered plants, blue light suppressed signalling of ABA-induced stomatal closure and promoted stomatal opening (Boccalandro et al., 2012). A recent publication demonstrated that growing chrysanthemum plants under red light reduced postharvest water loss from leaves. Possibly, this is caused by generation of small/fast acting stomata during the growth of plants (Seif et al., 2021). At this time, it is not understood how high relative humidity causes stomata to lose the ability to respond to closing stimuli (Aliniaeifard and van Meeteren, 2014; Arve et al., 2014). In rose, multiple genes involved in ABA pathway form a highly complex regulatory network acting together towards tolerance to high RH (Carvalho et al., 2016). Plants grown at high RH have reduced ABA content through upregulated ABA catabolism genes, or increased derivatization, however this does not entirely explain the reduced response to ABA (Schwartz et al., 2003; Lee et al., 2006; Rezaei Nejad and van Meeteren, 2006; Seki et al., 2007; Giday et al., 2013; Arve et al., 2015).

Several strategies are known to alleviate of stomatal malfunctioning in plants developed under high RH: increased salinity, ABA application, soil water deficit, increased air movement, grafting, increased blue light, temporary increase of temperature and temporary decrease of RH (Fanourakis et al., 2016). Some of those strategies could have a direct implication in increasing the vase life of cut roses (Fanourakis et al., 2013a).

## Carbohydrates

At harvest, cut flowers are excised from organs that are the source of carbohydrates: bulbs, tubers, roots, and stems. Cut flowers are required to metabolize and grow during the postharvest phase. Harvest and distribution often have a negative effect on the carbohydrate status of cut flowers. During transportation and storage, plant produce is usually kept in the dark in which photosynthesis is restricted, and the carbohydrate reserves are depleted by metabolic processes. Finally, during vase life, limited light, nutrient and water availability strongly impair photosynthesis, and carbohydrate availability is further reduced. These negative circumstances for maintaining carbohydrate reserves can strongly reduce postharvest quality of cut-flowers.

Carbohydrate starvation often leads to senescence-like symptoms, such as flower wilting, loss of chlorophyll in leaves and loss of chlorophyll and colour in flowers (van der Meulen-Muisers et al., 2001; Han, 2003; Ichimura et al., 2003; Buchanan-Wollaston et al., 2005; Kazuo et al., 2005; Trivellini et al., 2012; van Geest et al., 2016). However, carbohydrate starvation also leads to symptoms that are not observed during normal developmental senescence such as leaf blackening and reduced flower opening (Bieleski et al., 1992; van der Meulen-Muisers et al., 2001; Han, 2003; van Geest et al., 2016). The molecular pathways associated with developmental senescence and carbohydrate-starvation induced deterioration have many analogies, but there are also essential differences (Buchanan-Wollaston et al., 2005; Trivellini et al., 2012). It can therefore be difficult to separate postharvest problems associated with carbohydrate starvation or developmental senescence, because they might have the same initial trigger (van Doorn, 2004).

In chrysanthemum, cultivar differences in carbohydrate content of disk florets are related to susceptibility to disk floret degreening, with high light increasing the carbohydrate content of disk florets (van Geest et al., 2017). Choice of cultivar and light intensity can therefore significantly affect postharvest performance through carbohydrate content. Also altering the sink-source balance, by pruning, can strongly affect the amount of storage carbohydrates, and with that the postharvest performance (Zieslin et al., 1975; Kool et al., 1996).

Carbohydrates play an important role in the postharvest performance in cut-flowers; the addition of sugars to the vase water is usually one of the most effective measures to improve vase-life. Cut flowers that can better cope with carbohydrate starvation during the postharvest chain will be less perishable. This notion brings interesting opportunities to improve postharvest performance by breeding, improved growing practices and postharvest technology.

## Botrytis

*B. cinerea* (‘grey mould’) is a constant and costly threat to the ornamental industry with various attack modes, and the ability to survive in favourable and unfavourable conditions (Elad, 2016). Even some *B. cinerea* species that grow as harmless endophytes might turn into ‘necrotrophic thugs’ due to increased inbreeding and reduced genetic diversity in ornamental crops (van Kan et al., 2014). In addition, *B. cinerea* has the ability to quickly develop fungicide resistances (Li et al., 2014). *B. cinerea* isolates were found resistant to many single, and sometimes, several classes of fungicides in commercial rose shipments (Muñoz et al., 2019). Biological control agents such as fungi or bacteria are also alternatives as they disturb *B. cinerea* hyphae development and induce systemic resistance (Zhao et al., 2018; Calderón et al., 2019; Nakkeeran et al., 2020; South et al., 2020; Motlagh and Jafari, 2022).

Managing *B. cinerea* during ornamental production starts with proper sanitation protocols. Infections in cut roses has been correlated with the spore density in glasshouses with dead leaves as sources of inoculum (Kerssies et al., 1995; Dik and Wubben, 2007). The other key issue is to limit dew point temperatures to prevent free water on the crop by e.g., drip irrigation, reducing plant density and avoiding harvest on rainy days (Daughtrey and Benson, 2005; Elad, 2016). Fortification against *B. cinerea* is also a good strategy, by e.g., calcium sprays. Fortification against *B. cinerea* can also be elicited by phytohormones such as brassinosteroids, salicylic-, jasmonic- and abscisic acid (AbuQamar et al., 2017; Liu et al., 2018; Shafiee-Masouleh, 2018; Bennett et al., 2020). An interesting, additional tool, for *B. cinerea* management is the use of UV-C radiation. UV-C has shown to limit *B. cinerea* development in gerbera and freesia flowers (Darras et al., 2010). UV-C has recently been applied commercially in rose greenhouse cultivation as an end of day treatment and during sorting. It is likely a *B. cinerea* containment strategy can only be attained by both fortifying the ornamental crop and limiting the number of spores, either dormant or active, in a systemic management approach (Bika et al., 2021). Perhaps the toughest hurdle to overcome is that treatments published in literature are not necessarily effective in the ornamental industry due to ever changing cultivars, cultivation practises and global chains.

## Conclusions and future perspectives

The quality of cut flowers is difficult to predict because it is dependent on several interacting processes that depend on carbohydrate status, xylem architecture, stomatal behaviour, and microbial (botrytis) pressure; factors that are all shaped during cultivation. The question is whether the continuing accumulation of knowledge of important processes that shape quality is currently sufficient to provide a strategy to create the best conditions for the best cut flower quality. We mention a number of important factors; (1) good hygiene is important both for pathogenic and nonpathogenic microorganisms, (2) light conditions determine photosynthesis and the carbohydrate status, and (3) the importance for RH control is emphasized because high RH could lead to stomatal malfunction.. We feel that we are currently not able to do provide such a strategy and would like to emphasize gathering more information during the cultivation phase, for instance by embracing new phenotyping tools. For example, the influence of root substrate on xylem architecture is not well understood, and more research is needed. The use of novel non-destructive methods opens new possibilities in this topic. Recent developments in new phenotyping tools such as xylem architecture measurements with sound, and with 3D high-resolution X-ray micro-computed tomography (micro-CT) images have recently been reported (Wason et al., 2021; Dutta et al., 2022).

Complete control of growth conditions does not necessarily improve quality; vase life of zinnia grown in natural photoperiods from May to June was better than that of those grown under artificial long days in the greenhouse in February and April (Stimart and Brown, 1982). During field production, plants are grown under varying environmental conditions from spring to fall and this influences quality (Kalinowski et al., 2022). Dry periods can be good for xylem architecture, and high light improves carbohydrate deposition, although dynamic control of these factors is possible in protected horticulture.

Recent developments such as increases in gas prices and the implementation of LED lights to replace traditional HPS lamps in greenhouses can lead to changes in cut flower quality, especially in winter. Lower quality could be caused by a lower carbohydrate load, caused by a reduced photosynthesis, and reduced growth under lower temperatures. However, it is also possibly due to stomatal malfunctioning in plants developed under high RH. Strategies to prevent this, are difficult to implement in horticultural practices (e.g., ABA application is expensive) and therefore a more permanent solution is required. Identification of the molecular mechanism that leads to stomatal malfunction can lead to genetic markers that can be used to select for better stomatal function. A better understanding of optimal conditions for quality would allow for the application of dynamic growth conditions that yield this quality.

To study the complex effects of preharvest conditions on quality, illustrates the need for more rapid and reliable phenotyping methods. Hydration status might also be evaluated non-destructively by applying spectral information and artificial neural network. The water status at harvest of leaves of two ornamental species (*Spathiphyllum wallisii, Chrysanthemum morifolium*) was analysed by multi spectral imaging (Fanourakis et al., 2023). During growth, it might be possible to monitor stomatal function and crop health and performance using thermal cameras, or by spectral imaging (Stamford et al., 2023). Thermal camaras could be used to monitor stomatal behaviour and photosynthetic activity (Vialet-Chabrand and Lawson, 2020). In addition, currently, the development of complete controlled growth conditions in conditioned environmental agriculture and vertical farms, as well as monitoring of the plants during their growth has grown tremendously (van Delden et al., 2021). These high tech growth conditions offer the possibility to study the effect of growth conditions on postharvest quality in more detail (Rosenqvist et al., 2019; Hall et al., 2022).

## Author contributions

JV: Writing – original draft, Writing – review & editing. Wvl: Writing – original draft, Writing – review & editing. DC: Writing – original draft, Writing – review & editing. GvG: Writing – original draft, Writing – review & editing. RS: Writing – original draft, Writing – review & editing.
